# Supplementary material for: A Comprehensive Atlas of Cell Type Density Patterns and Their Role in Brain Organization
Source: bioRxiv. 2025 Mar 20:2024.10.02.615922. Preprint. [Version 2] doi: 10.1101/2024.10.02.615922 (PMC11956909; doi:10.1101/2024.10.02.615922)
Supplement: Supplement 1 — Supplementary Figure1: Cortical density distribution: a) STPT coronal sections showing the laminar distribution of nine cell type markers (VGlut1, SERT, Npy, Ntsr1, Emx, Crh, Calb, Rbp4, and Tlx3) across five cortical regions: frontal pole (FRP), primary motor cortex (MOp), primary somatosensory cortex (SSp), auditory cortex (AUD), and visual cortex (VIS). Red horizontal lines indicate cortical layer boundaries. b) Layer-based cortical flatmaps displaying the spatial distribution of the same nine cell types across four cortical layers (L2/3, L4, L5, L6). These flatmaps reveal both layer-specific and region-specific distribution patterns of each cell type marker. [file media-1.docx]

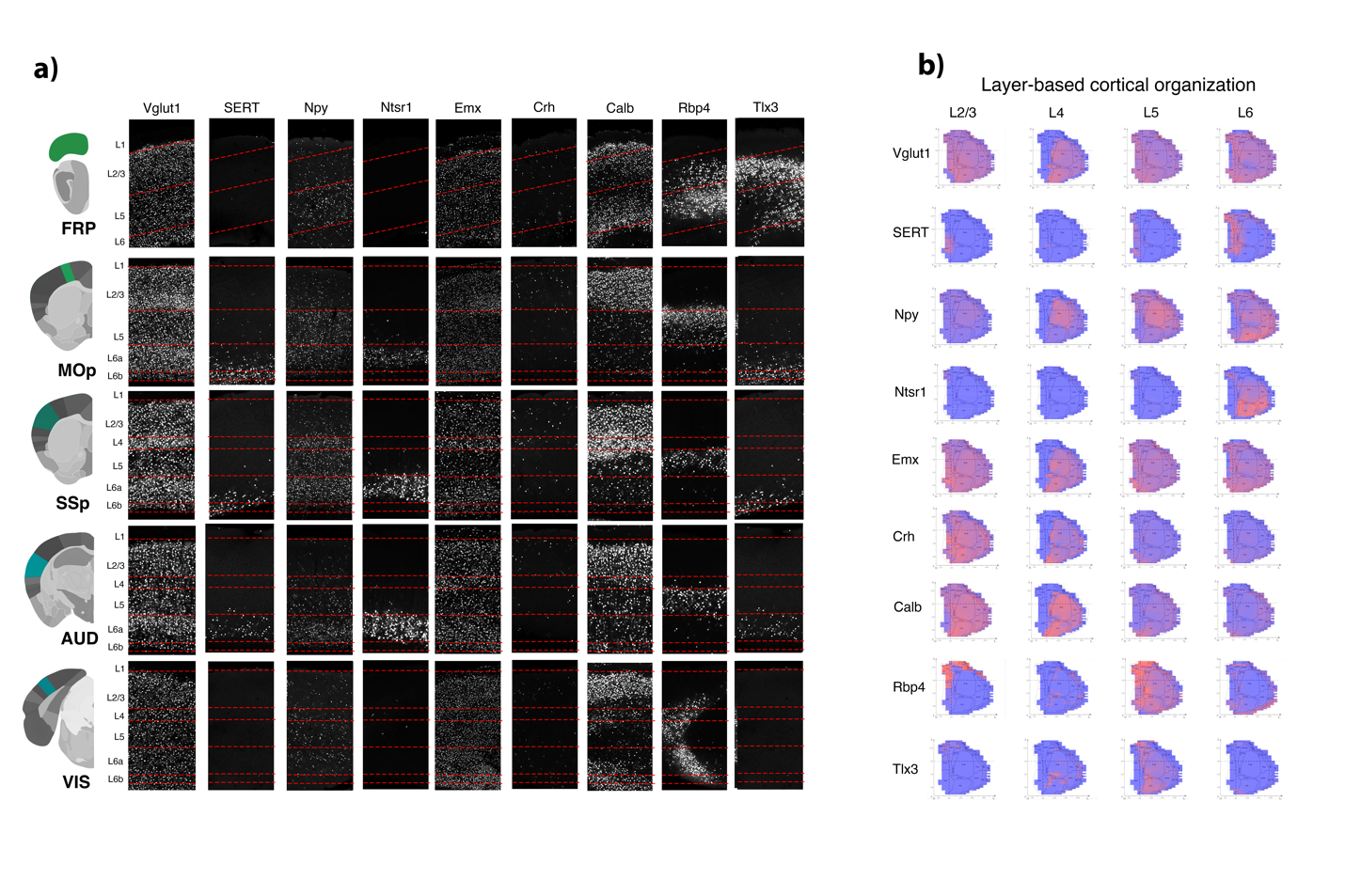


**Supplementary Figure2: Cortical density distribution: a)** STPT coronal sections showing the laminar distribution of nine cell type markers (VGlut1, SERT, Npy, Ntsr1, Emx, Crh, Calb, Rbp4, and Tlx3) across five cortical regions: frontal pole (FRP), primary motor cortex (MOp), primary somatosensory cortex (SSp), auditory cortex (AUD), and visual cortex (VIS). Red horizontal lines indicate cortical layer boundaries. **b)** Layer-based cortical flatmaps displaying the spatial distribution of the same nine cell types across four cortical layers (L2/3, L4, L5, L6). These flatmaps reveal both layer-specific and region-specific distribution patterns of each cell type marker.
