# Supplementary material for: A Comprehensive Atlas of Cell Type Density Patterns and Their Role in Brain Organization
Source: bioRxiv. 2025 Mar 20:2024.10.02.615922. Preprint. [Version 2] doi: 10.1101/2024.10.02.615922 (PMC11956909; doi:10.1101/2024.10.02.615922)
Supplement: Supplement 2 [file media-2.docx]

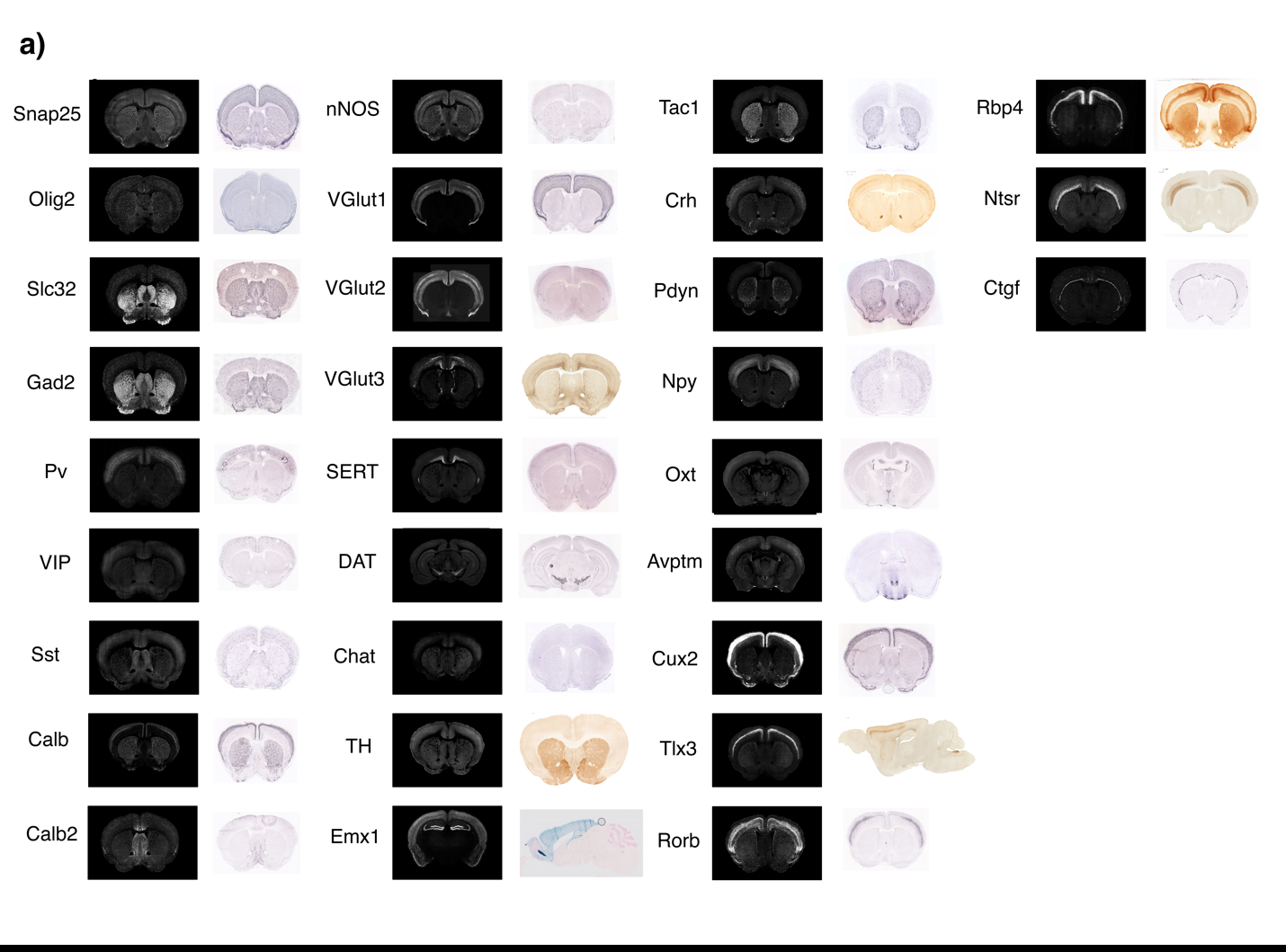


**Supplementary Figure1: Cre driver lines labeling validation.** STPT coronal planes (left panels) from the thirty Cre driver mice used in this study comparing the labeling to Allen Brain Atlas (ABA) *in situ* or GENSTAT data.
